# Penalized Variable Selection for Lipid–Environment Interactions in a Longitudinal Lipidomics Study

**DOI:** 10.3390/genes10121002

**Published:** 2019-12-03

**Authors:** Fei Zhou, Jie Ren, Gengxin Li, Yu Jiang, Xiaoxi Li, Weiqun Wang, Cen Wu

**Affiliations:** 1 Department of Statistics, Kansas State University, Manhattan, KS 66506, USA; 2 Department of Mathematics and Statistics, University of Michigan Dearborn, Dearborn, MI 48128, USA; 3 Division of Epidemiology, Biostatistics, and Environmental Health, School of Public Health, University of Memphis, Memphis, TN 38111, USA; 4 Department of Food, Nutrition, Dietetics and Health, Kansas State University, Manhattan, KS 66506, USA

**Keywords:** GEE, lipid–environment interaction, longitudinal lipidomics study, penalized variable selection

## Abstract

Lipid species are critical components of eukaryotic membranes. They play key roles in many biological processes such as signal transduction, cell homeostasis, and energy storage. Investigations of lipid–environment interactions, in addition to the lipid and environment main effects, have important implications in understanding the lipid metabolism and related changes in phenotype. In this study, we developed a novel penalized variable selection method to identify important lipid–environment interactions in a longitudinal lipidomics study. An efficient Newton–Raphson based algorithm was proposed within the generalized estimating equation (GEE) framework. We conducted extensive simulation studies to demonstrate the superior performance of our method over alternatives, in terms of both identification accuracy and prediction performance. As weight control via dietary calorie restriction and exercise has been demonstrated to prevent cancer in a variety of studies, analysis of the high-dimensional lipid datasets collected using 60 mice from the skin cancer prevention study identified meaningful markers that provide fresh insight into the underlying mechanism of cancer preventive effects.

## 1. Introduction

Longitudinal data are frequently observed in a diversity of scientific research areas, including economics, biomedical studies, and clinical trials. A common characteristic of the longitudinal data is that the same subject is measured repeatedly over a certain period of time; thus, the repeated measurements are correlated. Many modeling techniques have been proposed to accommodate the multivariate correlated nature of the data [1,2]. The emergence of new types of data has brought constant challenges to the development of novel statistical methods for longitudinal studies. One representative example is the high-dimensional data where the number of variables is much larger than the sample size. As penalization has been demonstrated as an effective way for conducting variable selection in linear and generalized linear models with a univariate response [3,4], substantial efforts have been devoted to developing penalized variable selection methods with longitudinal responses, such [5,6,7], among many others.

This study was partially motivated by overcoming the limitations of existing penalization methods in order to analyze the high-dimensional lipidomics data from longitudinal studies. Lipids are a broad group of biomolecules in eukaryotic membranes, involved in various critical biological roles such as energy storage, cellular membrane structure, or cell signaling and homeostasis [8,9,10,11]. Lipid metabolism has been found to be associated with several diseases, especially chronic diseases such as diabetes, cancer, inflammatory disease, and Alzheimer [12,13,14].

The lipid data were obtained from our previous work on the lipid changes in weight controlled CD-1 mice [15]. In the current study, the phenotype of interest is the body weight of experimental animals, which was measured every week for 10 weeks. The environmental factor was exercise and/or dietary restriction, which had four different levels, control (ad libitum feeding and sedentary), AE (exercise and ad libitum feeding), PE (exercise and pair feeding), and DCR (sedentary and 20% dietary calorie restriction). Both triacylglycerol (TG) and diacylglycerol (DG) profiles in the plasma were measured using electrospray ionization MS/MS [15]. Here, we focused on the DG profiles and treated them as lipid factors. Besides the lipid main effects, we were particularly interested in investigating the interactions between lipids and environment/treatment effects, which will shed novel insight in the understanding of weight changes in a longitudinal setting beyond studies solely focusing on the main lipidomics effects. With the control as the baseline, we created a group of three dummy variables to represent the four levels of the treatment factor that can be treated as environmental factors in general. The product between the dummy variable group and lipid denotes the lipid–environment interactions. The formulation of the interaction group in our study shared the spirit of group LASSO, which was primarily motivated by the selection of important dummy variable groups from ANOVA problems [16]. As the total number of main and interaction effects was much larger than the sample size, penalized variable selection was a natural choice to identify the important subset of effects. Such methods for G × E interactions, including [17,18], however, cannot be adopted for the longitudinal studies.

On the other hand, existing penalization methods in longitudinal studies have been mostly developed for the identification of important main effects only. For instance, Wang et al. [5] proposed the penalized generalized estimating equation (PGEE) to select predictors that are associated with the longitudinal response. Ma et al. [6] considered the selection of important predictors and estimation of non-parametric effects through splines for repeated measures data. Cho and Qu [7] developed a penalized quadratic inference function (PQIF) method to conduct variable selection on main effects. Fan et al. [19] developed robust variable selection through a penalized robust estimating equation to incorporate the correlation structure for repeated measurements. These studies have ignored the interaction effects and cannot be adopted to analyze our data directly. In addition, our limited search also suggests that user-friendly software packages for variable selection methods in longitudinal studies have been relatively underdeveloped. For penalization methods, only two R packages (PGEE and pgee.mixed) are available, and both packages have focused on the selection of important main effects. The codes for most studies in this area have not even been made publicly available.

To accommodate simultaneously the selection of individual and group structure corresponding to the main lipid effect and interaction effect respectively, we propose a novel penalized variable selection method for longitudinal clustered data. Our method significantly advances the existing penalization methods by considering the interaction effects. Through incorporating the group structure, selection of both main and interaction effects can be efficiently conducted within the generalized estimating equation framework [20]. Furthermore, to facilitate fast computation and reproducible research, we implement the proposed and benchmark methods in the R package (interep https://cran.r-project.org/package=interep) [21]. The software package is open-source, and the core module has been developed in C++. The advantage of our method over alternatives has been demonstrated in extensive simulation studies. Analysis of the motivating dataset yields findings with important implications.

## 2. Materials and Methods

### 2.1. Data and Model Settings

Consider a longitudinal study with *n* subjects and $k_{i}$ observations measured repeatedly across time for the *i*th subject (1⩽i⩽n). Without loss of generality, we set $k_{i}=k$. $Y_{ij}$ denotes the response for the *i*th subject at time *j* (1⩽j⩽k). $X_{ij}={\left(X_{ij1},\ldots ,X_{ijp}\right)}^{⊤}$ is the *p*-dimensional vector of lipid factors. In our study, $E_{ij}={\left(E_{ij1},\ldots ,E_{ijq}\right)}^{⊤}$ denotes the *q*-dimensional treatment factor. Suppose that the lipid factors, treatment factors, and their interactions are associated with the longitudinal phenotype through the following model:

(1) $$Y_{ij}=\beta_{0}+E_{ij}^{⊤}\beta_{1}+X_{ij}^{⊤}\beta_{2}+{\left(X_{ij}⊗E_{ij}\right)}^{⊤}\beta_{3}+\epsilon_{ij}=Z_{ij}^{⊤}\beta +\epsilon_{ij}$$

where $\beta ={\left(\beta_{0},\beta_{1}^{⊤},\beta_{2}^{⊤},\beta_{3}^{⊤}\right)}^{⊤}$ and $Z_{ij}=c(1,E_{ij}^{⊤},X_{ij}^{⊤},(X_{ij}⊗E_{ij}{)}^{⊤}{)}^{⊤}$ are (1+q+p+pq)-dimensional vectors that represent all the main and interaction effects. The interactions between lipids and treatment factors are modeled through $X_{ij}⊗E_{ij}$, the Kronecker product of the *p*-dimensional vector $X_{ij}$, and the *q*-dimensional vector $E_{ij}$ within the following form:

$$X_{ij}⊗E_{ij}={\left[X_{ij1}E_{ij1},X_{ij1}E_{ij2},\ldots ,X_{ij1}E_{ijq},X_{ij2}E_{ij1},\ldots ,X_{ijp}E_{ijq}\right]}^{⊤}$$

which is a pq-dimensional vector. $\beta_{0}$ is the intercept. $\beta_{1}$, $\beta_{2}$, and $\beta_{3}$ are unknown coefficient vectors of dimensions *q*, *p*, and pq, respectively. We assume that the observations are dependent within the same subject, and independent if they are from different subjects. $\epsilon_{i}={\left(\epsilon_{i1},\ldots ,\epsilon_{ik_{i}}\right)}^{T}$ follows a multivariate normal distribution $N_{k}\left(0,\Sigma_{i}\right)$, with $\Sigma_{i}$ as the covariance matrix for the repeated measure of the *i*th subject across the *k* time points.

### 2.2. Generalized Estimating Equations

The joint likelihood function for longitudinally clustered response $Y_{ij}$ is generally difficult to specify. Liang and Zeger [20] developed the generalized estimating equations (GEE) method to account for the intra-cluster correlation. It models the marginal instead of the conditional distribution given the previous observations and only requires a working correlation structure for $Y_{ij}$ to be specified.

The first two marginal moments of $Y_{ij}$ are denoted by $E\left(Y_{ij}\right)=\mu_{ij}=Z_{ij}^{T}\beta$ and $\mathrm{Var}\left(Y_{ij}\right)=\upsilon \left(\mu_{ij}\right)$, respectively, where υ is a known variance function. Then, the estimating equation for β is defined as:

(2) $$\sum_{i=1}^{n}\frac{\partial \mu_{i}\left(\beta \right)}{\partial \beta }V_{i}^{-1}\left(Y_{i}-\mu_{i}\left(\beta \right)\right)=0,$$

where $\mu_{i}\left(\beta \right)=(\mu_{i1}\left(\beta \right),\ldots ,\mu_{ik}\left(\beta \right){)}^{⊤}$, $Y_{i}=(Y_{i1},\ldots ,Y_{ik}{)}^{⊤}$ and $V_{i}$ is the covariance matrix of $Y_{i}$. The first term in (2), $\frac{\partial \mu_{i}\left(\beta \right)}{\partial \beta }$, reduces to $Z_{i}={\left(Z_{i1},\ldots ,Z_{ik}\right)}^{⊤}$, which corresponds to the k×(1+q+p+pq) matrix of the main and interaction effects.

$V_{i}$ is often unknown in practice and difficult to estimate especially when the number of variance components is large. In GEE, the covariance matrix $V_{i}$ is specified through a simplified working correlation matrix R(η) as $V_{i}=A_{i}^{\frac{1}{2}}R\left(\eta \right)A_{i}^{\frac{1}{2}}$, with the diagonal marginal variance matrix $A_{i}\;=\;\mathrm{diag}\left\{\mathrm{Var}\left(Y_{i1}\right),...,\mathrm{Var}\left(Y_{ik}\right)\right\}$. R(η) is characterized by a finite-dimensional nuisance parameter η. Commonly adopted correlation structures for R(η) can be independent, AR(1), and exchangeable, among others. Liang and Zeger [20] showed that if η can be consistently estimated, the GEE estimator of the regression coefficient is consistent. Furthermore, the consistency holds even when the working correlation structure is misspecified.

### 2.3. Penalized Identification

When the dimensionality of lipid factors is high, the total number of main and interaction effects is even higher. However, only a small subset of important effects is associated with the phenotype, which naturally leads to a variable selection problem. Penalized GEE based methods, including Wang et al. [5] and Ma et al. [6], have been proposed for conducting selection under correlated longitudinal responses. However, those published studies focus on the main effects and ignore the interactions. As shown in (1), the lipid–environment interactions are modeled on the group level, that is the interaction between all the *q* treatment factors and the *h*th lipidomics measurement (1⩽h⩽p). Such a group structure cannot be accommodated by variable selection methods from existing longitudinal studies. This fact motivates us to develop a method for the interaction analysis of repeated measures data, termed as interep, with the following penalized generalized estimating equation:

(3) $$Q\left(\beta \right)=U\left(\beta \right)-\sum_{g=1}^{p}\rho^{\prime }(|\beta_{2g}|;\lambda_{1},\gamma )\mathrm{sign}\left(\beta_{2g}\right)-\sum_{h=1}^{p}\rho^{\prime }(||\beta_{3h}{\left|\right|}_{\Sigma_{h}};\sqrt{q}\lambda_{2},\gamma ),$$

where U(β) is the score equation in GEE and $\rho^{\prime }(\cdot )$ is the first derivative of the minimax concave penalty (MCP) [22]. Since the environmental factors are usually of low dimension and are predetermined as important ones, they are not subject to penalized selection. U(β) is defined as:

$$U\left(\beta \right)=\sum_{i=1}^{n}Z_{i}^{T}V_{i}^{-1}\left(Y_{i}-\mu_{i}\left(\beta \right)\right),$$

and the MCP can be expressed as:

$$\rho \left(t;\lambda ,\gamma \right)=\lambda \int_{0}^{t}{\left(1-\frac{x}{\gamma \lambda }\right)}_{+}dx,$$

where λ is the tuning parameter and γ is the regularization parameter. The first derivative function of the MCP penalty is:

$$\rho^{\prime }\left(t;\lambda ,\gamma \right)=\left(\lambda -\frac{t}{\gamma }\right)I\left(t\leq \gamma \lambda \right).$$

MCP can be adopted for the regularized selection on both individual and group level effects. It is fast, continuous, and nearly unbiased [22].

In (3), the vector $\beta_{2}={\left(\beta_{21},\ldots ,\beta_{2p}\right)}^{⊤}$ denotes the regression parameters for all the *p* lipid factors. $\beta_{3}={\left(\beta_{31}^{⊤},\ldots ,\beta_{3p}^{⊤}\right)}^{⊤}$, which denotes the regression parameters for lipid–environment interactions, is a vector of length pq. $\beta_{3h}$ is a vector of length *q* (h=1,2,…,p), corresponding to the interactions between the *h*th lipid feature and the environment factors. With the control as the baseline, the environment factors have been formulated as a group of dummy variables. With high-dimensional main and interaction effects, penalization is critical for the identification of important effects out of the large number of candidates. In the penalized generalized estimating Equation (3), the first penalty term adopts MCP directly to conduct the selection of main lipid effects on the individual level. The second penalty, in the forms of group MCP, imposes shrinkage on the product between the lipid factors and dummy variable group, which corresponds to the lipid–environment interactions. The group level selection of interaction effects is consistent with the mechanism of creating the dummy variable group of environmental factors. Note that such a rationale of formulating the penalized generalized estimating Equation (3) is deeply rooted in group LASSO [16].

In particular, $\lambda_{1}$ and $\lambda_{2}$ in (3) are tuning parameters. $\rho^{\prime }(||\beta_{3h}{\left|\right|}_{\Sigma_{h}};\sqrt{q}\lambda_{2},\gamma )$ is the group MCP penalty that corresponds to the interactions between the *h*th (h=1,2,…,p) lipid factor and the *q* environment factors. The empirical norm $\left|\right|\beta_{3h}{\left|\right|}_{\Sigma_{h}}$ is defined as: $\left|\right|\beta_{3h}{\left|\right|}_{\Sigma_{h}}={\left({\beta^{\prime }}_{3h}\Sigma_{h}\beta_{3h}\right)}^{1/2}$ with $\Sigma_{h}=n^{-1}B_{h}^{⊤}B_{h}$. $B_{h}\;=\;Z\left[\left(2+q+p+\left(h-1\right)\times q\right):\left(1+q+p+h\times q\right)\right]$, and it contains the *q* columns in *Z* that correspond to the interactions from the *h*th lipid factor with the *q* environment factors.

A variety of penalized variable selection methods for high-dimensional longitudinal data have been developed in the past two decades for analyzing high-dimensional omics data, such as gene expressions, single nucleotide polymorphisms (SNPs), and copy number variations (CNVs) [5,6]. However, lipidomics data have been rarely investigated by using high-dimensional variable selection methods. We developed a package, (interep https://cran.r-project.org/package=interep) that incorporates our recently developed penalization procedures to conduct interaction analysis for high-dimensional lipidomics data with repeated measurements [21].

Remark: The uniqueness of the proposed study lies in accounting for the group structure of lipid–environment interactions through penalized identification. Therefore, the main lipid effects and lipid–environment interactions are penalized on individual and group levels, separately, which leads to a formulation of both MCP and group MCP penalties. Although our model has been motivated from a specific lipidomics profiling study in weight controlled mice [15], it can be readily extended to accommodate more general cases in interaction studies where the environmental factors are not dummy variables formulated from the ANOVA setting. In such a case, for each lipid factor, the main lipid effects and lipid–environment interactions form a group, with the leading component of the group being a vector of 1s. As not all the effects in the group are expected to be associated with the phenotype, a sparse group type of variable selection is demanded. Such a formulation has been investigated in survival analysis [23], but not in longitudinal studies yet. With a simple modification of our model to penalize the main and interaction effects on the individual and group level simultaneously, the proposed one becomes a penalized sparse group GEE model and can be adopted to handle general environmental factors in high-dimensional cancer genomics studies.

### 2.4. Computational Algorithms

We developed an efficient Newton–Raphson type of algorithm to obtain the penalized estimate $\hat{\beta }$. Starting with an initialized value, we can solve the penalized GEE iteratively. The estimated ${\hat{\beta }}^{\left(d+1\right)}$ in the (d+1)th iteration can be solved as:

(4) $${\hat{\beta }}^{\left(d+1\right)}={\hat{\beta }}^{\left(d\right)}+{\left[T^{\left(d\right)}+nW^{\left(d\right)}\right]}^{-1}\left[U^{\left(d\right)}-nW^{\left(d\right)}{\hat{\beta }}^{\left(d\right)}\right],$$

where $U^{\left(d\right)}$ is the score function expressed in terms of ${\hat{\beta }}^{\left(d\right)}$ at the *d*th iteration and $T^{\left(d\right)}$ is the corresponding first derivative function of $U^{\left(d\right)}$:

$$T^{\left(d\right)}=\sum_{i=1}^{n}Z_{i}^{T}V_{i}^{-1}Z_{i},$$

which is also a function of ${\hat{\beta }}^{\left(d\right)}$. The MCP penalty was imposed on both the individual level (main lipid effects) and group level (lipid–environment interactions). Therefore, $W^{\left(d\right)}$ is a diagonal matrix that contains the first derivative of the MCP penalty for the lipid factors and the first derivative of the group MCP penalty for the lipid–environment interactions. We define $W^{\left(d\right)}$ as:

$$\begin{array}{c} W^{\left(d\right)}=\mathrm{diag}\{\underset{1+q}{\underset{︸}{0,\ldots 0}},\frac{\rho^{\prime }\left(\right|{\hat{\beta }}_{21}^{\left(d\right)}\left|;\lambda_{1},\gamma \right)}{\epsilon +|{\hat{\beta }}_{21}^{\left(d\right)}|},\ldots ,\frac{\rho^{\prime }\left(\right|{\hat{\beta }}_{2p}^{\left(d\right)}\left|;\lambda_{1},\gamma \right)}{\epsilon +|{\hat{\beta }}_{2p}^{\left(d\right)}|},\\ \frac{\rho^{\prime }\left(|\right|{\hat{\beta }}_{31}^{\left(d\right)}{\left|\right|}_{\Sigma_{1}};\sqrt{q}\lambda_{2},\gamma )}{\epsilon +\left|\right|{\hat{\beta }}_{31}^{\left(d\right)}{\left|\right|}_{\Sigma_{1}}},\ldots ,\frac{\rho^{\prime }\left(|\right|{\hat{\beta }}_{3p}^{\left(d\right)}{\left|\right|}_{\Sigma_{p}};\sqrt{q}\lambda_{2},\gamma )}{\epsilon +\left|\right|{\hat{\beta }}_{3p}^{\left(d\right)}{\left|\right|}_{\Sigma_{p}}}\}, \end{array}$$

where ϵ is a small positive number set to $10^{-6}$ to avoid the numerical instability when the denominator is zero. The first (1 + q) elements on the diagonal of *W* are zero, suggesting that there is no shrinkage imposed on the coefficients for the intercept and the environmental factors. We can use $nW\hat{\beta }$ and nW to approximate the first derivative function of MCP in the penalized score equation and the second derivative function of the MCP penalty, respectively. Given a fixed tuning parameter, the regression parameter ${\hat{\beta }}^{\left(d+1\right)}$ can be updated iteratively till convergence. The stopping criterion is that the L1 norm for the L1 difference between two consecutive iterations is less than $10^{-3}$, and convergence can usually be achieved within 10 iterations.

There are two tuning parameters $\lambda_{1}$ and $\lambda_{2}$ and a regularization parameter γ. $\lambda_{1}$ controls the sparsity of lipid factors, and $\lambda_{2}$ determines sparsity among lipid–environment interactions. We chose the optimal tuning parameters $\lambda_{1}$ and $\lambda_{2}$ using five-fold cross-validation in both the simulation study and real data analysis. The regularization parameter γ was obtained via a data driven approach. In our numerical study, we examined a sequence of values, such as 1.8, 3, 4.5, 6, and 10, suggested by published studies, and found that the results were not sensitive to the choice of the value of γ, and then set the value at 3. We split the dataset into five equally sized subsets and took four of them as the training dataset, leaving the last subset as the testing dataset. The penalized estimates were obtained from the training data, and then, prediction performance was evaluated on the testing data. A joint search over a two-dimensional grid of ($\lambda_{1},\lambda_{2}$) was conducted to find the optimal pair of tuning parameters.

Given fixed tuning parameters, we implemented the algorithm as follows:

(1) Set the initial coefficient vector $\beta^{\left(0\right)}$ using LASSO;
(2) Update $\beta^{\left(d+1\right)}$ using Equation (4) at the (d+1)th iteration;
(3) Repeat Step (2) until the convergence criterion is satisfied.

In our study, we considered the methods considering both lipid main effects and lipid–environment interactions with exchangeable working correlation (A1), AR(1) working correlation (A2), and independence working correlation (A3). For comparison with the methods that cannot accommodate the identification of lipid–environment interactions, we also included A4–A6, which incorporate the exchangeable, AR(1), and independence working correlation, respectively. The alternative methods A4–A6 do not ignore the interaction effects. Instead, they treat the interaction effects individually, so the group structure considered in A1–A3 does not exist. We computed the CPU running time for 100 replicates of simulated lipidomics data with n=250,ρ=0.8,p=75 (with a total dimension of 304) and fixed tuning parameters on a regular laptop for A1–A6, which can be implemented using our developed package: (interep https://cran.r-project.org/package=interep) [21]. The CPU running time in seconds was 48.8 (A1), 40.2 (A2), 29.0 (A3), 49.3 (A4), 39.7 (A5), and 27.9 (A6), respectively.

## 3. Results

### 3.1. Simulation

We evaluated the performance of all six methods (A1–A6) through extensive simulation studies. Among them, A1–A3 were developed for accommodating the interaction structures with different working correlations, while A4–A6 were only focused on the identification of main effects so the structure of the group level interaction effects were not respected. Note that there are existing studies that can also achieve the selection of main effects in longitudinal studies. For example, Wang et al. [5] adopted the smoothly clipped absolute deviation (SCAD) penalty for conducting the selection of main effects. Since the MCP is incorporated as the baseline penalty in A1–A3, A4–A6 have thus been developed based on MCP and used as benchmark methods for comparison.

The responses were generated from the model (2) with sample size n= 250 and 500. The number of time points *k* was set to five. The dimensions for lipid factors $X_{ij}$ were *p* = 75, 150 and 300. With *q* = 3 for $E_{ij}$, we first simulated a vector of length *n* from the standard normal distribution. A group of three binary dummy variables for environmental factors could then be generated after dichotomizing the vector at the 30th and 70th percentiles. In addition, the lipids were simulated from a multivariate normal distribution with mean zero and the AR1 covariance matrix with marginal variance one and auto-correlation coefficient 0.5. We simulated the random error ϵ from a multivariate normal distribution by assuming a zero mean vector and an AR1 covariance structure with ρ= 0.5 and 0.8. Note that when considering the interactions, the actual dimensionality was much larger than *p*. For instance, given *n* = 250, *p* = 150, and *q* = 3, the total dimension for all the main and interaction effects was 604.

The coefficients were simulated from U[0.4,0.8] for 17 nonzero effects, consisting of the intercept, 3 environmental dummy variables, 4 lipid main effects, and 3 groups of lipid–environment interactions (9 interaction effects). We generated 100 replicates for the four settings: (1) *n* = 250 and *p* = 75, (2) *n* = 250 and *p* = 150, (3) *n* = 500 and *p* = 150, and (4) *n* = 500 and *p* = 300. All the rest of the coefficients were set to zero. For each setting, we considered two correlation coefficients (ρ= 0.5 and 0.8) for the random error. The number of true positives (TP) and false positives (FP) was recorded.

In addition to identification results, we also calculated the estimation accuracy in terms of the difference between estimated and true coefficients. In particular, the mean squared error corresponding to the true nonzero coefficients and true zero coefficients (for noisy effects) were termed as MSE and NMSE, respectively. The total mean squared error for the coefficient vector, or TMSE, is computed as:

$$\mathrm{TMSE}=\frac{1}{100}\sum_{r=1}^{100}\left|\right|{\hat{\beta }}^{\left(r\right)}-\beta {\left|\right|}^{2}/p_{\beta }$$

where $p_{\beta }$ is the dimension of β and ${\hat{\beta }}^{\left(r\right)}$ is the estimated value of β in the *r*th simulated dataset. MSE and NMSE were calculated in a similar way as for TMSE.

**Figure 1:**
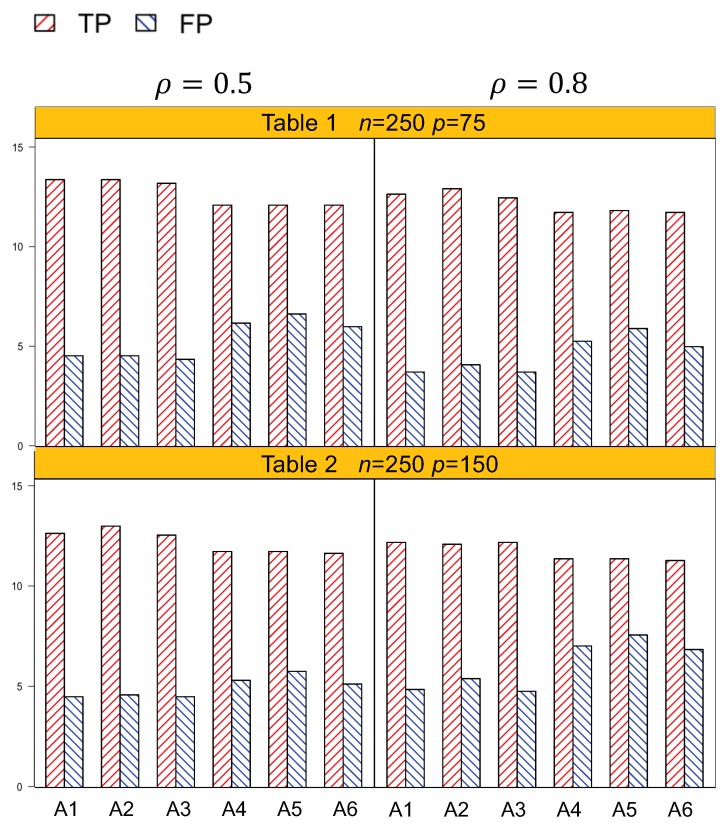
Plot of the identification results for n=250, p=75 with an actual dimension of 304. p=150 with an actual dimension of 604. A1–A3: methods accommodating the lipid–environment interactions with exchangeable, AR(1), and independence working correlations, respectively. A4–A6: methods not accommodating the lipid–environment interactions with exchangeable, AR(1), and independence working correlations, respectively.

**Figure 2:**
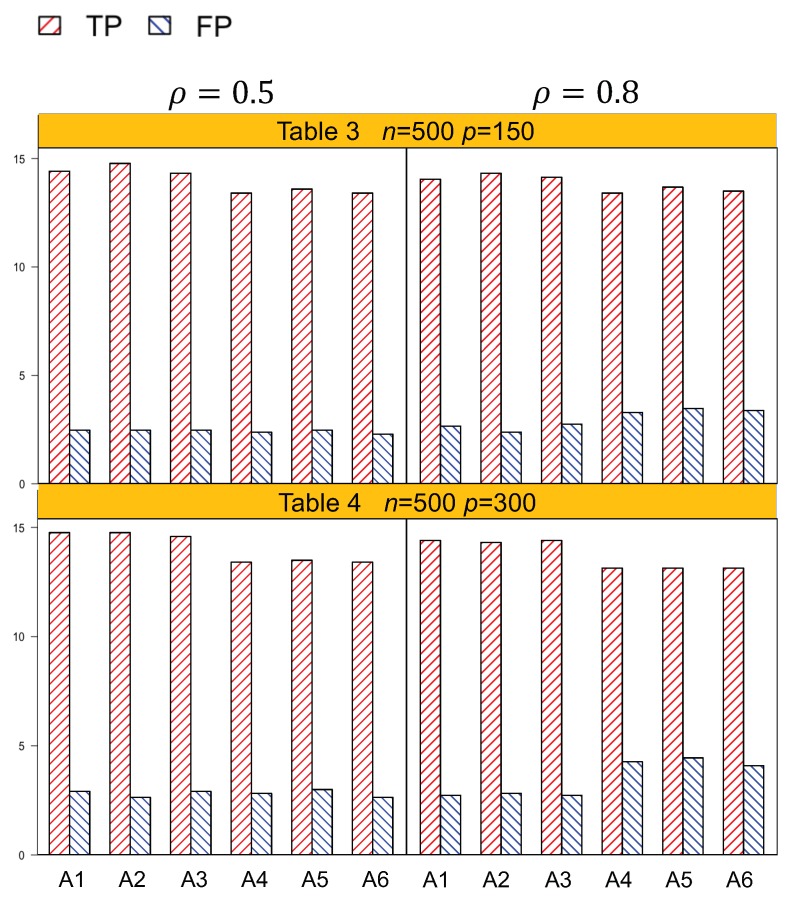
Plot of the identification results for n=500, p=150 with an actual dimension of 604. p=300 with an actual dimension of 1204. A1–A3: methods accommodating the lipid–environment interactions with exchangeable, AR(1), and independence working correlations, respectively. A4–A6: methods not accommodating the lipid–environment interactions with exchangeable, AR(1), and independence working correlations, respectively.

Identification results of the six methods (A1–A6) are tabulated in Table 1, Table 2, Table 3 and Table 4. In general, A1–A3, which account for both the lipid main effects and lipid–environment interactions, had better performance than A4–A6, which only accommodated the main effects. For example, in Table 1, given *n* = 250, ρ=0.5, *p* = 75, the actual dimension is 304. A1 identified 14.5 (sd 1.9) nonzero effects out of all the 17 true positives, with a relatively small number of false positives of 4.8 (sd 3.1). On the other hand, A4 identified a smaller number of true positives, 1.3 (sd 1.5), with a larger number of false positives, 6.6 (sd 4.2). Among the identified effects, A1 identified 7.4 (sd 1.5) interactions, with 3.1 (sd 2.6) false positives. A4 identified a smaller TP of 6.1 (sd 1.1) and a higher FP of 5.1 (sd 3.3) of the lipid–environment interactions. We could observe that the difference in identification performance between A1 and A4 came mainly from the interaction effects, which was due to the fact that A4 could not accommodate the group level selection corresponding to the lipid–environment interactions. As the dimension increased, A1 outperformed A4 more significantly. For instance, in Table 4, the overall dimension for *n* = 500, ρ=0.8, *p* = 300 is 1204. A1 had a TP of 15.9 (sd 1.2) and an FP of 3 (sd 2.6), while A4 had a smaller TP 14.5 (sd 1.2) and a higher FP 4.5 (sd 3.0). Figure 1 and Figure 2 are plotted based on the identification results from Table 1, Table 2, Table 3 and Table 4. We can observe that overall, A1–A3 outperformed A4–A6 with a higher TP and a lower FP under each setting.

In terms of estimation accuracy, A1–A3 also had a better performance compared with A4–A4, as shown in Table 5 and Table 6. For the panel corresponding to *n* = 250, ρ=0.5, and *p* = 75 in Table 5, the mean squared error for the nonzero coefficients of A1 was 0.1055, which was less than half of that of A4 (0.2321). Besides, A1 also had a smaller total mean squared error (TMSE). All the pieces of evidence suggested that A1 had higher estimation accuracy than A4. We can observe the pattern for the rest of the four methods. As the dimension increased to *n* = 500, ρ=0.8, and *p* = 300 (so the total dimension was 1204) in Table 6, the MSE of A1 (0.0688) was also smaller than that of A4 (0.1949). There were no obvious differences in NMSE among these settings.

Another important conclusion we make from the simulation study is that, for the methods that differ only in working correlation, i.e., A1 (exchangeable), A2 (AR1), and A3 (independence), there was no significant difference in terms of either identification or estimation accuracy, as shown by Table 1, Table 2, Table 3, Table 4, Table 5 and Table 6, as well as Figure 1 and Figure 2. Such an observation suggests that the proposed methods under the GEE framework were robust to the misspecification of the working correlation, and this is consistent with the conclusions from main effects only models in longitudinal studies [7].

To mimic the sample size and number of lipid factors in the case study, we also conducted a simulation in settings with *n* = 60, *p* = 30, and *q* = 3. Therefore, the overall dimension of main and interaction effects was 124. The coefficients were generated from *U*[1.4,1.8] for 17 nonzero effects. The identification and prediction results are summarized in Table A1 and Table A2 in the Appendix A, respectively. Consistent patterns were observed. For example, in terms of identification, under ρ = 0.5, A1 had a higher TP of 13.6 (sd 2.5) compared to the 11.1 (sd 2.6) of A4, and a lower FP of 4.7 (sd 2.7), compared to the FP of 5.4 (sd 2.8) identified by A4.

Evaluation of all the methods, especially A1–A3, was also conducted when the true underlying model was misspecified. We generated the response (phenotype) from a main effect only model with eight true main effects when *n* = 250, *p* = 75, ρ=0.8 with a total dimension of 304. Results are provided in Table A3. When the interaction effects did not exist, A1 had only identified a very small number of false interaction effects, with 0.7 (sd 1.7) false positives. A2–A6 performed similarly in terms of identifying false interaction effects. All six methods identified a comparable number of true main effects. Overall, all methods had similar performance in identification, as well as prediction, when the data generating model had only main effects. Such a phenomenon is reasonable by further examining the results in Table 1. We found that the major difference between A1–A3 and A4–A6 was due to the identification of interaction effects. Therefore, when only main effects were present, all the methods had comparable performances.

Penalized regression and hypothesis testing are two related, but distinct aspects in statistical analysis. The proposed study was not aimed at developing test statistics, computing the power functions, and assessing the control of type 1 error, so these statistical test related results are not available, just like most of the studies on penalized regression. Recently, efforts devoted to bridging the two areas have been mainly restricted to linear models under high-dimensional settings [24,25,26]. Extensions to interaction models have not been reported so far. In particular, we are not aware of results reported for longitudinal models. Nevertheless, we conducted the simulation by assuming the null model and tabulate the identification results in Table A4. The results should be interpreted as identification with misspecified models. As we observed, under the null model, all six methods led to a very small number of false positives.

To assess the consistency of variable selection in longitudinal settings, we carried out the stability selection [27] under *n* = 250, *p* = 75, and ρ = 0.8. Each time, we selected 200 out of the total of 250 subjects without replacement and then conducted selection. The process was repeated 100 times, which yielded a proportion of selected effects. Larger proportions of being selected suggested stable results. Stability selection is well known for assessing the stability of penalized selection, and it alleviates the concern that the effects have only been identified by chance. We investigate the selection proportions of the 17 true main and interaction effects for all six methods in Table A5. A1 identified 14 true effects with proportions above 70%, which is consistent with the results shown in the lower panel of Table 1, where 13.7 TPs (sd 2.3) were identified. Such a consistent pattern can be observed across all six methods.

Although no consensus on the optimal criterion of selecting tuning parameters has been reached so far, cross-validation is perhaps the most well accepted criterion to select tuning parameters in the community of high-dimensional data analysis [3,4]. To further justify its appropriateness, under the setting of *n* = 250 and *p* = 75, we performed the analysis by selecting tuning parameters using an independently generated testing dataset with a sample size of 1000 and *p* = 75. The models were fitted on the training dataset, and prediction was assessed based on the independently generated testing dataset, so no data were used in training the model. The identification and prediction results are tabulated in Table A6 and Table A7, respectively. A comparison to Table 1 and Table 5 demonstrates that the results obtained by cross-validation and validation were very close.

### 3.2. Real Data Analysis

We applied the proposed and alternative methods on a dataset from one of our previous studies in animal models [15]. In the study, 60 female CD-1 mice were assigned to four different treatment groups, which were control (ad libitum feeding and sedentary), AE (exercise and ad libitum feeding), PE (exercise and pair feeding), and DCR (sedentary and 20% dietary calorie restriction). The phenotype of interest was mice’s body weight, which was measured every week for 10 weeks. Mice were sedentary and given ad libitum feeding in the control group, where they could eat as much as they wanted without doing treadmill exercises. In the AE group, mice received ad libitum feeding and ran on the treadmill every day at a speed of 0.5 mph, 1 h per day, and 5 days a week, while mice in the PE group did the same exercise, but were given the same amount of diet as the mice in the control group. Mice in the DCR group had 20% less calorie intake than the control group, but they had the same intake of protein, vitamins, and minerals. The composition of 176 plasma neutral lipid species of interest was measured. In the current study, we only focused on diacylglycerols. In addition, the diacylglycerol lipid species that have a majority of samples lower than the detection limits were excluded so there were 31 diacylglycerols. In total, there were 31 lipid main effects and 93 lipid–environment interactions.

Using the method A1 (interep with the exchangeable working correlation) as shown in Table 7, we identified seven lipid species that had different effects in weight control of mice (AE, PE, or DCR) on body weight compared to those of the control mice. Among them, C20:1/16:1 and C20:1/20:4 had negative interactions in AE mice, where C denotes carbon. For the lipid species of C20:1/16:1, $C_{39}H_{76}O_{5}N$, the regression coefficient was −2.9145 for AE mice. That is, mice with an increased amount of C20:1/16:1 tended to have a lower body weight compared to that of the control. In the AE mice, both C16:0/C16:0 and C22:6/C18:1 had strong positive associations with body weights. It is interesting that C16:0/C16:0 were negatively associated with body weight in both PE and DCR mice. C16:0 is also called palmitic acid and is one of most common saturated fatty acids. Increased consumption of palmitic acid is associated with higher risk of cardiovascular disease, type 2 diabetes, and cancer [28]. The negative association of C16:0/16:0 and body weight in DCR and PE suggests that when the calories of the diet are restricted, the accumulation of saturated fat in the body actually decreased compared to the control. Another lipid that is negatively associated with body weight in DCR and PE mice is C18:1/16:1. The lipids that were positively associated with body weight in PE were C18:2/C16:1, C20:1/C16:1, and C22:6/C18:1. All species contain unsaturated fatty acids. Among them, C22:6 is one of the omega-3 polyunsaturated fatty acids (PUFA). In DCR, the two lipids that were positively associated with body weight were C18:2/16:1 and C20:1/20:4. Both fatty acids C18:2 and C20:4 were PUFA. The results seem to be consistent with our previous finding that exercise with paired feeding may increase the amount of PUFA in phospholipids in mice skin [29].

In addition, we adopted A4 to analyze the lipid data. A4 also had the exchangeable working correlation, but it could not conduct group level selection of the lipid–environment interactions. The identification results are tabulated in Table 8. Note that the selection of interactions with individual dummy environment factors was not consistent with the formulation of the lipid–environment interactions. In terms of prediction, A1 had a smaller prediction error (4.04) than that of A4 (4.97).

## 4. Discussion

Investigation of the potential roles of lipids in the regulation and control of cellular function and the interactions between lipids and environmental factors are very important in the understanding of physiology and disease processes. Traditionally, the analyses mostly focus on the total amount of a particular type of lipid, such as total triglyceride, total cholesterol, and omega-3 fatty acid. With the recent advances in instrumental technology, it is feasible to analyze quantitatively a broad range of lipid species in a single platform [13,15,30,31,32]. The vast arrays of data generated in lipid profiling studies bring challenges to the statistical analysis of lipidomics data [33,34,35].

In this study, we proposed a penalized variable selection method to identify important lipid–environmental effects in longitudinal studies. Some statistical methods have already been reported for lipidomics studies, including the marginal test and variable selection methods [15,32,34,35]; however, they cannot be directly extended to longitudinal studies. On the other hand, existing variable selection methods for longitudinal data have been predominately developed for the identification of main effects and cannot accommodate the group level interaction structure unique to our studies. Both the simulation and case study have convincingly demonstrated the merit of the proposed interep over alternatives.

We selected tuning parameters based on cross-validation. A further investigation of different tuning criteria is interesting, but beyond the scope of this study, especially given the fact that many well known variable selection methods in longitudinal studies, such as [5], have been conducted using cross-validation. To facilitate a fair cross-comparison with existing relevant studies, we believe it is reasonable to adopt cross-validation to choose tuning parameters. Note that the aforementioned stability selection analysis also partially justifies the usage of cross-validation. We acknowledge that other criteria for selecting tunings, such as double cross-validation [36], could be a potential reliable choice. However, as it is not a widely accepted tuning criterion for high-dimensional data analysis and has not been adopted in any longitudinal studies so far, we postpone the investigation to the future.

Interaction studies have been historically pursued by statisticians [37]. Within the high-dimensional scenario, accounting for such a complex structure, in both gene–gene (G × G) and gene–environment (G × E) interaction studies, is challenging, but also rewarding [38]. The proposed study is among the first to investigate penalized identification of lipid–environment interactions in longitudinal studies. Both the simulation study and case study yielded interesting findings. G × G interaction is computationally more challenging than G × E interactions since both main effects involved in the interactions are of high dimensionality. Following the representative G × G interaction studies [39,40], we can extend the proposed study to lipid–lipid interactions, which has not been investigated in longitudinal studies so far. Besides, when multi-omics measurements are available, it is also of great interest to examine interaction effects through multi-omics integration studies in the longitudinal setting [41,42].

The proposed model can also be estimated using the quadratic inference functions (QIF). GEE relies on the working correlation matrix R(η), and it enables us to find the consistent estimator of the regression parameter if consistent estimators of the nuisance parameters η can be obtained. However, consistent estimators of η do not always exist in some cases. QIF has been proposed to avoid explicit estimation of the nuisance parameters by assuming the inverse of the working correlation matrix R(η) can be approximated by a linear combination of a class of base matrices [7,43]. Thus, QIF is robust to the misspecification of the working correlation.

In this paper, we are interested in the identification of lipid-treatment (or environment) interactions through penalization. The success of set based analysis, including those for the gene set [44] and SNP set [45,46], has tremendously motivated the development of statistical methods for G × E interactions from marginal analyses ([47,48]) to penalization methods [17,18,49]. Our model can be potentially extended in the following aspects. First, as data contamination and outliers have been widely observed in repeated measurements, robust variable selection methods in G × E interaction studies [23,50,51,52] can be extended to longitudinal settings. Second, recently, multiple Bayesian methods have been proposed for pinpointing important G × E interaction effects [53,54,55]. Within the framework of analyzing repeated measurements, Bayesian variable selection for interactions has not been extensively examined. Investigations of all these possible directions will be postponed to the near future.

## Figures and Tables

**Table 1:** Identification results for n=250, p=75 with an actual dimension of 304.

n=250	p=75	Overall		Main		Interaction	
		TP	FP	TP	FP	TP	FP
ρ = 0.5	A1	14.5(1.9)	4.8(3.1)	7.2(0.8)	1.7(1.2)	7.4(1.5)	3.1(2.6)
	A2	14.7(1.8)	5.0(3.2)	7.2(0.9)	1.7(1.3)	7.5(1.4)	3.2(2.6)
	A3	14.7(1.7)	5.0(3.3)	7.2(0.8)	1.8(1.4)	7.6(1.3)	3.2(2.6)
	A4	13.3(1.5)	6.6(4.2)	7.2(0.7)	1.6(1.4)	6.1(1.1)	5.1(3.3)
	A5	13.3(1.5)	6.8(4.4)	7.2(0.8)	1.7(1.4)	6.1(1.1)	5.2(3.5)
	A6	13.3(1.5)	7.3(4.7)	7.2(0.8)	1.8(1.5)	6.1(1.1)	5.5(3.7)
ρ = 0.8	A1	13.7(2.3)	4.1(2.8)	7.2(0.8)	1.5(1.0)	6.5(2.1)	2.7(2.4)
	A2	13.9(2.4)	4.1(2.8)	7.2(0.8)	1.5(1.0)	6.6(2.1)	2.7(2.4)
	A3	14.2(2.3)	4.5(2.9)	7.2(0.7)	1.6(1.0)	7.0(2.2)	2.9(2.5)
	A4	12.9(1.9)	5.5(2.7)	7.2(0.7)	1.1(1.0)	5.6(1.6)	4.5(2.3)
	A5	12.9(1.9)	5.8(2.9)	7.2(0.7)	1.1(0.9)	5.7(1.6)	4.7(2.5)
	A6	13.0(1.8)	6.5(3.5)	7.2(0.7)	1.2(0.9)	5.8(1.4)	5.5(3.2)

Mean (sd) based on 100 replicates. A1–A3: methods accommodating the lipid–environment interactions with exchangeable, AR(1), and independence working correlations, respectively. A4–A6: methods not accommodating the lipid–environment interactions with exchangeable, AR(1), and independence working correlations, respectively.

**Table 2:** Identification results for n=250, p=150 with an actual dimension of 604.

n=250	p=150	Overall		Main		Interaction	
		TP	FP	TP	FP	TP	FP
ρ = 0.5	A1	13.9(2.3)	5.0(3.0)	7.2(0.7)	1.7(1.1)	6.7(2.0)	3.3(2.6)
	A2	14.0(2.2)	5.0(3.0)	7.2(0.7)	1.7(1.1)	6.8(1.9)	3.3(2.6)
	A3	14.4(2.2)	5.1(3.2)	7.3(0.7)	1.8(1.2)	7.1(1.9)	3.3(2.8)
	A4	12.9(1.9)	5.7(2.5)	7.3(0.8)	1.4(0.9)	5.6(1.5)	4.4(2.3)
	A5	13.0(1.8)	5.9(2.6)	7.2(0.8)	1.4(0.9)	5.7(1.4)	4.5(2.3)
	A6	13.0(1.8)	6.4(2.7)	7.2(0.8)	1.4(1.0)	5.8(1.5)	5.0(2.5)
ρ = 0.8	A1	13.5(2.0)	5.3(3.0)	7.2(0.9)	2.1(1.2)	6.3(1.9)	3.2(2.4)
	A2	13.5(2.0)	5.4(3.2)	7.2(0.9)	2.2(1.3)	6.3(1.9)	3.2(2.5)
	A3	13.4(2.1)	6.0(3.0)	7.1(0.9)	2.4(1.3)	6.2(1.9)	3.6(2.7)
	A4	12.5(1.9)	7.6(3.3)	7.3(0.7)	1.8(1.2)	5.2(1.7)	5.7(2.7)
	A5	12.6(1.8)	7.8(3.4)	7.3(0.7)	1.9(1.2)	5.3(1.6)	5.9(2.8)
	A6	12.6(1.8)	8.4(4.1)	7.3(0.8)	1.9(1.2)	5.4(1.7)	6.5(3.6)

Mean (sd) based on 100 replicates. A1–A3: methods accommodating the lipid–environment interactions with exchangeable, AR(1), and independence working correlations, respectively. A4–A6: methods not accommodating the lipid–environment interactions with exchangeable, AR(1), and independence working correlations, respectively.

**Table 3:** Identification results for n=500, p=150 with an actual dimension of 604.

n=500	p=150	Overall		Main		Interaction	
		TP	FP	TP	FP	TP	FP
ρ = 0.5	A1	15.7(1.4)	2.7(1.9)	7.7(0.5)	1.3(0.7)	8.0(1.4)	1.4(1.7)
	A2	15.8(1.3)	2.7(2)	7.7(0.5)	1.3(0.7)	8.1(1.3)	1.3(1.8)
	A3	16.2(1.2)	2.7(1.9)	7.8(0.4)	1.3(0.8)	8.4(1.2)	1.3(1.6)
	A4	14.7(1.0)	2.5(1.7)	7.8(0.4)	0.9(0.8)	6.9(1.0)	1.6(1.4)
	A5	14.7(1.1)	2.6(1.7)	7.8(0.4)	0.9(0.7)	6.9(1.0)	1.7(1.4)
	A6	14.9(1.0)	2.7(2.0)	7.8(0.4)	0.8(0.7)	7.0(0.9)	1.8(1.6)
ρ = 0.8	A1	15.5(1.7)	3.0(2.9)	7.7(0.6)	1.1(0.8)	7.9(1.5)	1.9(2.2)
	A2	15.4(1.7)	2.9(2.8)	7.7(0.6)	1.1(0.8)	7.8(1.5)	1.8(2.2)
	A3	15.7(1.6)	2.6(2.6)	7.7(0.5)	1.2(0.9)	8.0(1.4)	1.4(2.1)
	A4	14.8(1.4)	3.7(1.8)	7.5(0.6)	1.2(0.7)	7.2(1.2)	2.5(1.5)
	A5	14.7(1.3)	3.6(1.9)	7.5(0.5)	1.1(0.7)	7.2(1.2)	2.5(1.5)
	A6	15.0(1.3)	3.8(1.9)	7.7(0.6)	1.1(0.7)	7.4(1.1)	2.7(1.6)

Mean (sd) based on 100 replicates. A1–A3: methods accommodating the lipid–environment interactions with exchangeable, AR(1), and independence working correlations, respectively. A4–A6: methods not accommodating the lipid–environment interactions with exchangeable, AR(1), and independence working correlations, respectively.

**Table 4:** Identification results for n=500, p=300 with an actual dimension of 1204.

n=500	p=300	Overall		Main		Interaction	
		TP	FP	TP	FP	TP	FP
ρ = 0.5	A1	16.1(1.2)	3.2(2.4)	7.6(0.6)	1.4(0.8)	8.5(1.0)	1.8(2.2)
	A2	16.3(1.1)	3.2(2.4)	7.7(0.5)	1.4(0.8)	8.5(0.9)	1.8(2.2)
	A3	16.3(1)	2.9(2.2)	7.8(0.5)	1.4(0.8)	8.6(0.8)	1.5(1.9)
	A4	14.8(0.8)	2.9(2.1)	7.8(0.4)	1.0(0.8)	7.0(0.8)	1.9(1.7)
	A5	14.8(0.9)	3.1(2.3)	7.8(0.4)	1.0(0.8)	7.0(0.8)	2.0(1.9)
	A6	14.9(0.9)	3.3(2.6)	7.8(0.4)	1.0(0.8)	7.1(0.9)	2.3(2.1)
ρ = 0.8	A1	15.9(1.2)	3(2.6)	7.6(0.5)	1.5(0.8)	8.3(1.1)	1.5(2.2)
	A2	15.9(1.3)	3.0(2.7)	7.6(0.5)	1.5(0.9)	8.2(1.1)	1.5(2.2)
	A3	15.8(1.4)	3.1(2.8)	7.7(0.5)	1.6(1.0)	8.1(1.2)	1.6(2.2)
	A4	14.5(1.2)	4.5(3.0)	7.8(0.6)	1.0(0.7)	6.8(1.0)	3.5(2.6)
	A5	14.5(1.2)	4.7(3.3)	7.8(0.6)	1.1(0.8)	6.7(0.9)	3.6(2.9)
	A6	14.5(1.1)	4.9(3.6)	7.8(0.6)	1.0(0.8)	6.7(0.8)	3.8(3.3)

Mean (sd) based on 100 replicates. A1–A3: methods accommodating the lipid–environment interactions with exchangeable, AR(1), and independence working correlations, respectively. A4–A6: methods not accommodating the lipid–environment interactions with exchangeable, AR(1), and independence working correlations, respectively.

**Table 5:** Estimation accuracy results for n=250, p=75 with an actual dimension of 304. p=150 with an actual dimension of 604.

		n=250					
		p=75			p=150		
		MSE	NMSE	TMSE	MSE	NMSE	TMSE
ρ = 0.5	A1	0.1055	0.0026	0.0043	0.1264	0.0045	0.0072
	A2	0.1042	0.0026	0.0042	0.1259	0.0045	0.0072
	A3	0.1030	0.0026	0.0042	0.1174	0.0041	0.0066
	A4	0.2321	0.0018	0.0056	0.2435	0.0032	0.0084
	A5	0.2304	0.0018	0.0055	0.2402	0.0031	0.0082
	A6	0.2288	0.0018	0.0055	0.2346	0.0030	0.0080
ρ = 0.8	A1	0.1187	0.0087	0.0135	0.129	0.0048	0.0075
	A2	0.1163	0.0085	0.0132	0.1295	0.0048	0.0075
	A3	0.1066	0.0075	0.0118	0.1319	0.0049	0.0077
	A4	0.2410	0.0060	0.0162	0.2531	0.0038	0.0092
	A5	0.2426	0.0060	0.0162	0.2487	0.0038	0.0091
	A6	0.2335	0.0058	0.0157	0.2431	0.0037	0.0089

Mean (sd) based on 100 replicates. A1–A3: methods accommodating the lipid–environment interactions with exchangeable, AR(1), and independence working correlations, respectively. A4–A6: methods not accommodating the lipid–environment interactions with exchangeable, AR(1), and independence working correlations, respectively.

**Table 6:** Estimation accuracy results for n=500, p=150 with an actual dimension of 604. p=300 with an actual dimension of 1204.

		n=500					
		p=150			p=300		
		MSE	NMSE	TMSE	MSE	NMSE	TMSE
ρ = 0.5	A1	0.0754	0.0026	0.0042	0.0660	0.0010	0.0017
	A2	0.0731	0.0026	0.0041	0.0659	0.0010	0.0017
	A3	0.0648	0.0022	0.0035	0.0663	0.0010	0.0017
	A4	0.1872	0.0015	0.0055	0.1635	0.0007	0.0024
	A5	0.1837	0.0015	0.0054	0.1612	0.0007	0.0024
	A6	0.1792	0.0013	0.0052	0.1603	0.0007	0.0024
ρ = 0.8	A1	0.0708	0.0023	0.0037	0.0688	0.0010	0.0018
	A2	0.0716	0.0023	0.0038	0.0688	0.0011	0.0018
	A3	0.0704	0.0025	0.0039	0.0718	0.0012	0.0020
	A4	0.1480	0.0013	0.0049	0.1949	0.0007	0.0028
	A5	0.1492	0.0013	0.0045	0.1945	0.0007	0.0028
	A6	0.1479	0.0012	0.0044	0.1899	0.0007	0.0027

Mean (sd) based on 100 replicates. A1–A3: methods accommodating the lipid–environment interactions with exchangeable, AR(1), and independence working correlations, respectively. A4–A6: methods not accommodating the lipid–environment interactions with exchangeable, AR(1), and independence working correlations, respectively.

**Table 7:** Real data analysis result from method A1 (method accommodating the lipid–environment interactions with exchangeable working correlation).

	Lipid	AE	PE	DCR
C16:0/16:1	0	0.0117	−0.0239	−0.0057
C18:2/16:1	0	0.1544	3.3322	0.3924
C18:1/16:1	0	0.4857	−0.6299	−0.5559
C20:1/16:1	0.5966	−2.9145	0.1299	−1.4836
C16:0/16:0	0	1.3742	−0.8817	−1.8070
C20:6/16:0	0.0369	0	0	0
C20:0/18:3	−1.3628	0	0	0
C18:0/18:2	−1.6154	0	0	0
C22:6/18:1	1.1717	1.7526	0.2287	−0.4079
C18:2/20:4	1.1497	0	0	0
C18:1/20:4	0.8490	0	0	0
C20:1/20:4	0	−0.2169	−0.6096	3.0537

AE, exercise and ad libitum feeding; PE, exercise and pair feeding; DCR, sedentary and 20% dietary calorie restriction.

**Table 8:** Real data analysis result from method A4 (method not accommodating the lipid–environment interactions with exchangeable working correlation).

	Lipid	AE	DCR	PE
C16:0/16:1	0	0	−0.0024	0
C18:2/16:1	−2.1856	0	3.2306	0
C18:1/16:1	0	0	−1.4641	−2.3563
C20:1/16:1	0.0042	−2.6768	0	−1.7757
C16:0/16:0	0	2.8757	−0.9389	−2.6791
C18:2/16:0	0	0	0	−1.7688
C20:6/16:0	0.1481	−0.1276	0	0
C18:1/18:3	0	0	1.2917	0
C20:0/18:3	−1.6171	0	0	0
C18:0/18:2	−1.7695	0	0	0
C22:6/18:1	0.8851	3.4714	0.4809	0
C18:1/18:0	0	−1.2901	0	0
C22:7/18:0	0	−0.9839	0	0
C18:2/20:4	2.5871	0.6150	0	1.9327
C18:1/20:4	0	0	−0.0031	0
C20:1/20:4	0.7542	−1.1147	0	3.5396

## Abbreviations

The following abbreviations are used in this manuscript:

GEE	Generalized estimating equation
AE	Exercise and ad libitum feeding
PE	Exercise and pair feeding
DCR	Sedentary and 20% dietary calorie restriction
TG	Triacylglycerol
DG	Diacylglycerol
LASSO	Least absolute shrinkage and selection operator
PGEE	Penalized generalized estimating equation
PQIF	Penalized quadratic inference function
MCP	Minimax concave penalty
SCAD	Smoothly clipped absolute deviation
SNP	Single nucleotide polymorphisms
CNV	Copy number variations
QIF	Quadratic inference function

## Appendix A

**Table A1:** Identification results for n=60, p=30 with an actual dimension of 124.

n=60	p=30	Overall		Main		Interaction	
		TP	FP	TP	FP	TP	FP
ρ = 0.5	A1	13.6(2.5)	4.7(2.7)	7.4(0.8)	2.1(1.6)	6.2(2.1)	2.5(2.6)
	A2	13.6(2.5)	4.8(2.8)	7.3(0.8)	2.2(1.6)	6.2(2.1)	2.6(2.6)
	A3	13.7(2.5)	4.9(3.0)	7.4(0.7)	2.1(1.6)	6.3(2.1)	2.7(2.7)
	A4	11.1(2.6)	5.4(2.8)	6.4(1.1)	1.1(1.0)	4.6(1.9)	4.3(2.3)
	A5	11.1(2.6)	5.4(2.8)	6.4(1.1)	1.1(1.0)	4.6(1.9)	4.3(2.3)
	A6	11.1(2.5)	5.5(2.8)	6.5(1.2)	1.1(1.0)	4.7(1.8)	4.4(2.3)
ρ = 0.8	A1	13.2(2.2)	4.4(2.9)	7.5(0.6)	2.4(1.7)	5.7(2.1)	1.9(2.1)
	A2	13.2(2.2)	4.4(2.9)	7.5(0.6)	2.4(1.7)	5.7(2.1)	2.0(2.1)
	A3	13.4(2.0)	4.4(3.0)	7.5(0.6)	2.4(1.7)	5.9(1.9)	2.0(2.1)
	A4	11.0(2.4)	5.5(2.5)	6.5(1.4)	1.3(1.2)	4.5(1.8)	4.2(2.1)
	A5	11.0(2.4)	5.6(2.6)	6.5(1.4)	1.3(1.2)	4.5(1.8)	4.2(2.2)
	A6	11.1(2.4)	5.8(2.7)	6.5(1.4)	1.4(1.3)	4.5(1.8)	4.3(2.2)

Mean (sd) based on 100 replicates. A1–A3: methods accommodating the lipid–environment interactions with exchangeable, AR(1), and independence working correlations, respectively. A4–A6: methods not accommodating the lipid–environment interactions with exchangeable, AR(1), and independence working correlations, respectively.

**Table A2:** Estimation accuracy results for n=60, p=30 with an actual dimension of 124.

	n=60,p=30					
	ρ=0.5			ρ=0.8		
	MSE	NMSE	TMSE	MSE	NMSE	TMSE
A1	0.9352	0.1928	0.2732	0.9820	0.2108	0.2944
A2	0.9387	0.1924	0.2733	0.9809	0.2105	0.2940
A3	0.9324	0.1914	0.2717	1.0098	0.2063	0.2933
A4	1.9732	0.1560	0.3528	1.9910	0.1488	0.3484
A5	1.9709	0.1556	0.3523	1.9887	0.1487	0.348
A6	1.9629	0.1543	0.3502	1.9795	0.1474	0.3458

Mean (sd) based on 100 replicates. A1–A3: methods accommodating the lipid–environment interactions with exchangeable, AR(1), and independence working correlations, respectively. A4–A6: methods not accommodating the lipid–environment interactions with exchangeable, AR(1), and independence working correlations, respectively.

**Table A3:** Data simulated based on the underlying main effect only model. Identification results for n=250,p=75,ρ=0.8 with an actual dimension of 304.

	Overall		Main		Interaction				
	TP	FP	TP	FP	TP	FP	MSE	NMSE	TMSE
A1	7.7(0.9)	0.7(1.7)	7.7(0.9)	0.0(0.0)	0.0(0.0)	0.7(1.7)	0.1025	0.0000	0.0014
A2	7.8(0.6)	0.4(1.3)	7.8(0.6)	0.0(0.2)	0.0(0.0)	0.4(1.3)	0.0730	0.0000	0.0010
A3	7.9(0.3)	0.5(1.2)	7.9(0.3)	0.3(0.7)	0.0(0.0)	0.2(0.8)	0.0288	0.0000	0.0004
A4	7.3(1.1)	0.8(0.9)	7.3(1.1)	0.0(0.0)	0.0(0.0)	0.8(0.9)	0.2530	0.0000	0.0034
A5	7.2(1.1)	0.9(1.1)	7.2(1.1)	0.0(0.0)	0.0(0.0)	0.9(1.1)	0.2273	0.0001	0.0031
A6	7.5(0.7)	1.2(1.1)	7.5(0.7)	0.0(0.2)	0.0(0.0)	1.2(1.1)	0.1932	0.0001	0.0027

Mean (sd) based on 100 replicates. A1–A3: methods accommodating the lipid–environment interactions with exchangeable, AR(1), and independence working correlations, respectively. A4–A6: methods not accommodating the lipid–environment interactions with exchangeable, AR(1), and independence working correlations, respectively.

**Table A4:** Null models.

	n=250				n=500			
	p=75		p=150		p=150		p=300	
	ρ=0.5	ρ=0.8	ρ=0.5	ρ=0.8	ρ=0.5	ρ=0.8	ρ=0.5	ρ=0.8
A1	0.00(0.00)	0.03(0.18)	0.03(0.18)	0.00(0.00)	0.00(0.00)	0.00(0.00)	0.00(0.00)	0.00(0.00)
A2	0.03(0.10)	0.03(0.18)	0.30(0.70)	0.10(0.31)	0.00(0.00)	0.00(0.00)	0.03(0.18)	0.00(0.00)
A3	0.13(0.51)	0.17(0.44)	0.97(1.47)	0.77(0.81)	0.10(0.40)	0.50(0.20)	0.10(0.31)	0.10(0.25)
A4	0.00(0.00)	0.03(0.18)	0.03(0.18)	0.00(0.00)	0.00(0.00)	0.00(0.00)	0.00(0.00)	0.00(0.00)
A5	0.03(0.10)	0.03(0.18)	0.30(0.70)	0.10(0.31)	0.00(0.00)	0.00(0.00)	0.03(0.18)	0.00(0.00)
A6	0.13(0.51)	0.17(0.44)	0.97(1.47)	0.77(0.81)	0.10(0.40)	0.50(0.20)	0.10(0.31)	0.10(0.25)

Mean (sd) based on 100 replicates. A1–A3: methods accommodating the lipid–environment interactions with exchangeable, AR(1), and independence working correlations, respectively. A4–A6: methods not accommodating the lipid–environment interactions with exchangeable, AR(1), and independence working correlations, respectively.

**Table A5:** Stability selection percentages for all 17 true effects in the simulated data when n=250, p=75, ρ=0.8 with an actual dimension of 304.

True Effect	A1	A2	A3	A4	A5	A6
1	1	1	1	1	1	1
2	0.73	1	1	0.82	0.98	1
3	1	0.80	1	1	1	1
4	1	1	1	1	1	1
5	1	0.45	1	1	0.93	0.98
6	0.13	0.14	0.38	0.65	0.98	0.98
7	0.58	0.65	1	0.99	1	0.92
8	0.61	0.25	0.45	0.89	1	1
9	1	0.84	1	0.46	0.02	0.10
10	1	0.86	1	0.07	0.01	0.10
11	1	0.83	1	0.7	0.66	0.84
12	0.77	0.91	0.72	0.36	0.87	0.01
13	0.77	0.91	0.73	0.39	0.94	0.45
14	0.75	0.94	0.77	0.48	1	0.98
15	0.81	0.82	0.98	0.30	0.55	1
16	0.80	0.86	0.99	0.98	0.75	0.99
17	0.80	0.87	0.99	0.66	0.93	1

A1–A3: methods accommodating the lipid–environment interactions with exchangeable, AR(1), and independence working correlations, respectively. A4–A6: methods not accommodating the lipid–environment interactions with exchangeable, AR(1), and independence working correlations, respectively.

**Table A6:** Validation methods. Identification results for n=250, p=75 with an actual dimension of 304.

n=250	p=75	Overall		Main		Interaction	
		TP	FP	TP	FP	TP	FP
ρ = 0.5	A1	14.1(2.1)	4.6(3.1)	7.0(0.8)	1.1(0.8)	7.0(1.8)	3.5(2.9)
	A2	14.2(2.1)	4.7(3.1)	7.0(0.9)	1.1(0.9)	7.1(1.8)	3.6(2.8)
	A3	14.4(1.7)	4.6(3.2)	7.1(0.8)	1.1(0.9)	7.2(1.5)	3.5(3.0)
	A4	13.1(1.1)	6.1(2.8)	6.9(0.8)	1.0(0.8)	6.1(0.9)	5.3(2.6)
	A5	13.1(1.1)	6.4(2.8)	6.9(0.8)	1.0(0.8)	6.1(0.9)	5.6(2.5)
	A6	13.0(1.2)	6.7(3.1)	6.9(0.8)	1.0(0.8)	6.1(1.0)	5.9(2.9)
ρ = 0.8	A1	13.7(2.6)	4.7(2.9)	7.2(0.8)	1.4(0.9)	6.5(2.3)	3.2(2.5)
	A2	13.8(2.6)	4.6(3.1)	7.3(0.8)	1.4(1.0)	6.6(2.3)	3.1(2.6)
	A3	13.8(2.5)	5.1(3.0)	7.3(0.7)	1.5(0.8)	6.5(2.1)	3.6(2.9)
	A4	12.9(2.1)	5.7(2.5)	7.3(0.8)	1.3(0.9)	5.6(1.6)	4.5(2.1)
	A5	12.9(2.1)	5.8(2.6)	7.3(0.8)	1.3(1.0)	5.6(1.6)	4.5(2.2)
	A6	12.9(2.2)	6.8(2.7)	7.3(0.7)	1.4(0.9)	5.6(1.8)	5.5(2.5)

Mean (sd) based on 100 replicates. A1–A3: methods accommodating the lipid–environment interactions with exchangeable, AR(1), and independence working correlations, respectively. A4–A6: methods not accommodating the lipid–environment interactions with exchangeable, AR(1), and independence working correlations, respectively.

**Table A7:** Validation methods. Estimation accuracy results for n=250, p=75 with an actual dimension of 304.

	n=250,p=75					
	ρ=0.5			ρ=0.8		
	MSE	NMSE	TMSE	MSE	NMSE	TMSE
A1	0.1126	0.0074	0.0120	0.1205	0.0085	0.0134
A2	0.1095	0.0071	0.0115	0.1200	0.0085	0.0133
A3	0.1082	0.0071	0.0115	0.1245	0.0090	0.0140
A4	0.2344	0.0051	0.0150	0.2610	0.0060	0.0171
A5	0.2335	0.0050	0.0149	0.2627	0.0060	0.0171
A6	0.2302	0.0048	0.0146	0.2565	0.0058	0.0166

Mean (sd) based on 100 replicates. A1–A3: methods accommodating the lipid–environment interactions with exchangeable, AR(1), and independence working correlations, respectively. A4–A6: methods not accommodating the lipid–environment interactions with exchangeable, AR(1), and independence working correlations, respectively.
